# Apoptosis of mural granulosa cells is increased in women with diminished ovarian reserve

**DOI:** 10.1007/s10815-019-01446-5

**Published:** 2019-04-13

**Authors:** Yuting Fan, Yajie Chang, Lina Wei, Jianhui Chen, Jingjie Li, Sierra Goldsmith, Sherman Silber, Xiaoyan Liang

**Affiliations:** 1 Reproductive Medical Center, The Sixth Affiliated Hospital of Sun Yat-sen University, 17th Shou-gou-ling Rd, Guangzhou, 510655 China; 2 Infertility Center of St. Louis, 224 S. Woods Mill Road Suite 730, St. Louis, MO 63017 USA

**Keywords:** Mural granulosa cells, Cumulus cells, Apoptosis, Oocyte developmental competence, Clinical outcomes

## Abstract

**Purpose:**

To evaluate the relationship between apoptosis of granulosa cells in women with normal ovarian reserve versus diminished ovarian reserve, and relate that to follicular fluid hormones, and to clinical outcomes.

**Methods:**

A prospective cohort study was initiated between October 2015 and June 2016 involving a total of 164 women undergoing IVF/ICSI cycles at a single IVF center. Mural and cumulus granulosa cells, and follicularfluid were collected during oocyte retrieval. Annexin V-FITC/PI apoptosis staining and flow cytometryanalysis were performed to evaluate apoptosis rate of mural granulosa cells and cumulus cells. Follicularfluid hormones were measured by ECLIA. Laboratory and clinical outcomes were analyzed.

**Results:**

In mural granulosa cells, early, late and total apoptosis rates were significantly increased in women with diminished ovarian reserve when compare to women with normal ovarian reserve, along with lower AMHand progesterone levels (but higher estradiol levels) in follicular fluid. Early apoptosis rate of cumulus cellswas significantly higher in the non-pregnant group. The apoptosis rate of mural cells was negativelycorrelated with parameters related to ovarian response, oocyte yield, MII egg number, 2pn cleavagenumber, D3 good embryos number, blastocyst formation rate and frozen embryos number. A positivecorrelation was found between mural granulosa cell apoptosis and age.

**Conclusion:**

A significantly higher apoptosis rate of mural granulosa cells was correlated with worse ovarian response, with fewer egg and embryo numbers in IVF/ICSI, as well as with age. Early apoptosis rate of cumulus cellsmight also have influence on clinical pregnancy.

**Electronic supplementary material:**

The online version of this article (10.1007/s10815-019-01446-5) contains supplementary material, which is available to authorized users.

## Introduction

Ovarian reserve is a complex clinical phenomenon influenced by age, genetics, autoimmune, and environmental factors [1]. It has been used to describe a woman’s reproductive potential and to predict response to controlled ovarian hyperstimulation in clinical practice. Diminished ovarian reserve defined by a reduced quantity and quality of oocytes represents a major challenge in assisted reproductive treatment (ART) [2–5]. However, the etiological and pathological mechanisms of diminished ovarian reserve are likely multifactorial and still poorly understood [6].

Apoptosis is an ongoing process during different stages of follicular development [7]. Animal experiments indicate that there are specific spatiotemporal patterns of apoptosis regulating oocyte maturation and follicle growth. The disorder of apoptosis of granulosa cells may influence cell connection between granulosa cells themselves and between granulosa cells and the oocyte [8]. There are two kinds of granulosa cells within the follicle according to their location and the connectivity with the oocyte from the antral follicle stage. The cumulus granulosa cells envelop the oocyte and have cross-talk with the oocyte across the zona pellucida, while the mural granulosa cells are separated from the oocyte by the cumulus cells and by the antral cavity filled with follicular fluid. Both the mural granulosa cells and the cumulus cells produce and secrete steroidal hormones as well as growth factors into the follicular fluid [9].

Higher apoptosis of granulosa cells has been associated with a higher percentage of empty follicles, fewer oocytes retrieved, and poorer quality of oocytes and embryos. Apoptosis of granulosa cells has been reported to have a negative effect on pregnancy rate and live birth rate with in vitro fertilization (IVF) [10, 11]. An increase of cumulus cell apoptosis has been reported in patients over 40 years old [12]. However, apoptosis in granulosa cells has been shown not to be related to follicular quality, oocyte maturity, or fertilizability in ICSI [13]. A recent study indicates that there is less apoptosis of granulosa cells in women with diminished ovarian reserve [14]. Thus, it is still controversial whether apoptosis of granulosa cells has an influence on oocyte quality and ovarian response. The majority of these studies focused on only granulosa cells isolated from follicular fluid during oocyte retrieval. Even though mural granulosa cells and cumulus cells begin in primordial, primary, secondary stage as a single-cell type, during folliculogenesis, especially during antral follicle and preovulatory follicle, they express different gene expression profiles [15]. A recent study on micro-RNAs shows that there are different expression profiles in mural granulosa cells rather than cumulus cells of young women with diminished ovarian reserve [16]. Apoptosis of mural granulosa cells and cumulus cells may have a different influence in women with normal ovarian reserve versus those with diminished ovarian reserve.

In this paper, we report a prospective cohort study to investigate multiple factors in the relationship between apoptosis of mural granulosa cells and cumulus cells, with clinical outcomes of patients with normal ovarian reserve and diminished ovarian reserve undergoing in vitro fertilization/intracytoplasmic sperm injection ((IVF/ICSI). Also, in order to better define the correlation between granulosa cell apoptosis and follicular fluid steroidal hormones, we analyzed the levels of steroids, including anti-Műllerian hormone (AMH), estradiol (E2), luteinizing hormone (LH), progesterone (P), and testosterone (T) in follicular fluid.

## Materials and methods

### Patients

The design of this prospective cohort study was approved by the IRB at Sixth Affiliated hospital of Sun Yat-sen University. Patients undergoing egg retrievals at the Center of Reproductive Medicine, Sixth Affiliated hospital were consented for collection and use of their discarded follicular fluid and granulosa cells for research purposes. A total of 164 women aged 21–46 years undergoing IVF/ICSI from October 2015 to June 2016 were included in this study. Enrolled patients met the following inclusion criteria: (i) adequate visualization of the ovaries on transvaginal ultrasound scans, (ii) no endocrine disorders and or history of ovarian surgery affecting the ovaries or gonadotropin/sex steroid secretion, and (iii) no current hormone therapy. Primary outcome was clinical pregnancy rate defined as a positive fetal heart activity on 12-week ultrasound. We further defined this patient population as two subgroups, normal ovarian reserve and diminished ovarian reserve. Diminished ovarian reserve was defined using a modification of the Bologna criteria. Patients with two or more of the following were classified as diminished ovarian reserve: (1) basal serum AMH < 2 ng/mL, day 3 follicle-stimulating hormone (FSH) ˃ 10 IU/L, and antral follicle count ≤ 6. There were 118 patients in the normal ovarian reserve group and 46 in the diminished ovarian reserve group.

### Basic hormonal measurements and antral follicle count measurement

Whole blood specimens were collected on menstrual day 3. Basic serum AMH, estradiol (E_2_), luteinizing hormone (LH), progesterone (P_4_), and testosterone (T) levels were measured by electrochemiluminescence immunoassay (ECLIA) (Roche Diagnostics GmbH, Mannheim, Germany). Antral follicle count was recorded with transvaginal ultrasound in both ovaries by measuring all visible follicles ≥ 3 mm in diameter; cystic morphologies larger than 12 mm were excluded.

### Stimulation protocol

Patients were assigned to long protocols, antagonist protocols, or minimal stimulation protocols based on the patient’s age, cause of infertility, basal AMH/FSH level, antral follicle count, previous ovarian response to gonadotropin, body mass index, and physician preference.

For long protocols, pituitary downregulation via administration of a gonadotrophin-releasing hormone agonist (GnRH-a) (Decapeptyl; Ipsen Pharmaceuticals, France) was performed in the mid-luteal phase before stimulation cycle. After 14 days, ovarian stimulation was started using recombinant FSH (Gonal-F; Merck Serono, Switzerland) injection. The starting dose varied from 150 to 375 IU based on patient’s age, AMH, antral follicle count, and BMI. Doses were further adjusted according to ovarian response, which was periodically monitored with transvaginal ultrasound and serum E2 and FSH.

For antagonist protocols, ovarian stimulation was started using 150 IU to 375 IU recombinant FSH (Gonal-F, Merk Serono, Switzerland) on day 3 of menstrual cycle. Follicle growth was monitored by transvaginal ultrasound, and serum E2, FSH, and LH levels were tested periodically. A dose of 0.25 mg GnRH-antagonist, cetrorelix (Cetrotide, Merck Serono; or Orgalutran, MSD) was initiated on day 6 of the cycle or when the leading follicle reached 14 mm in diameter. GnRH-antagonist was injected subcutaneously daily until hCG trigger.

For minimal stimulation protocols, patients were given 50 mg of clomiphene citrate or 2.5 mg of letrozole for 5 days. FSH of 75–150 IU (Gonal-F; Merck Serono, Switzerland) was injected every 2 days, and GnRH-antagonist was injected subcutaneously daily when the leading follicle reached 14 mm in diameter until hCG trigger.

The patients were administered 6000–10,000 IU of hCG when the leading follicle reached 18 mm in diameter with at least three follicles > 16 mm in diameter. Ultrasound-guided transvaginal oocyte retrieval was performed 34–36 h after hCG administration.

### Mural granulosa cells, cumulus cells, and follicular fluid collection

Mural granulosa cells, cumulus cells, and follicular fluid were obtained via ultrasound-guided transvaginal oocyte retrieval, which was performed 34–36 h after hCG injection. Aspirate from the first punctured follicle was used to collect cumulus cells and follicular fluid. Cumulus cells were manually trimmed from the cumulus-oocyte complex by the embryologist. Cumulus cells and follicular fluid were transferred to round–bottom polystyrene tubes separately. The oocyte was transferred to the fertilization dish and continued through standard embryology lab procedures for either IVF or ICSI and embryo culture. The follicular aspiration from the rest of follicles (except the first punctured follicle), after removal of oocyte-cumulus complexes, was pooled and stored in 50-mL conical polypropylene tubes (BD Falcon, Becton Dickinson, Franklin Lakes, NJ) for mural granulosa cell isolation.

Mural granulosa cells, cumulus cells, and follicular fluid were transferred to the lab for further processing in 1 h. Mural granulosa cells were isolated from follicular aspirates using Ficoll methods. Follicular fluids that contained mural granulosa cells were washed three times with cold sterile phosphate-buffered saline (PBS) at 500 g for 5 min at room temperature. Four milliliters of PBS was added to pellets and diluted solution was layered carefully on 6 mL of Ficoll-Paque (GE Healthcare, Uppsala, Sweden). The samples were centrifuged at 1000*g* for 25 min at room temperature. The cells that were in the interface were collected for apoptosis staining. Follicular fluid was centrifuged at 2500*g* for 10 min, and the supernatants were transferred to 1.5-mL microcentrifuge tube and stored at − 80 °C.

### Annexin V-FITC/PI apoptosis staining and flow cytometry

**Fig. 1:**
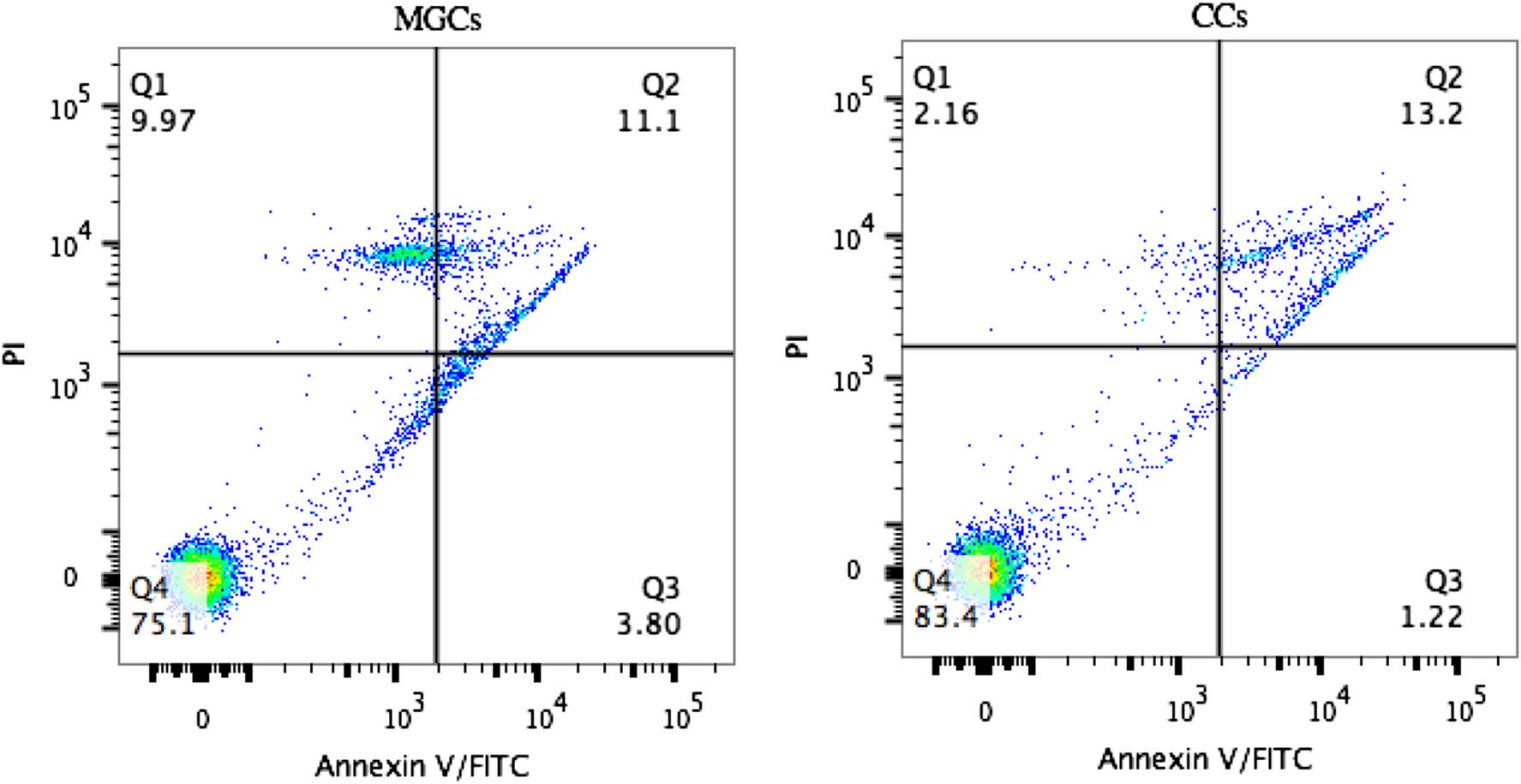
Annexin V-FITC/PI staining

To determine apoptosis of mural granulosa cells and cumulus cells, FITC Annexin V-FITC/PI double staining was evaluated using the FITC Annexin V Apoptosis Detection Kit (BD Biosciences, San Diego, CA). Single-cell suspensions of mural granulosa cells and cumulus cells were washed twice with cold PBS and then resuspended in 1× binding buffer at a concentration of 1 × 10^6 cells/mL. Specimens were stained for 15 min with Annexin V-FITC and propidium iodide (PI) at room temperature in the dark and analyzed by flow cytometry (BD Bioscience FacsCanto II, San Diego, CA) within 1 h. At least 10,000 cells were analyzed per patient. The percentage distribution of viable, early apoptotic, late apoptotic, and necrotic cells was calculated using FlowJo v10 (Fig. 1).

### Intrafollicular hormone assay

Follicular fluid was thawed and equilibrated to room temperature for 30 min and mixed well before analysis. Follicular fluid AMH, E2, P_4_, T were measured by ECLIA (Roche Diagnostics GmbH, Mannheim, Germany) as previously described.

### Embryo transfer and luteal phase support

The retrieved oocytes were fertilized by IVF or ICSI. Fresh transfers were performed either on D3 or D5 based on patients’ preference and physician’s suggestion. We only included the first frozen embryo transfer (FET) cycle if the patient did not perform fresh transfer in this oocyte retrieval cycle. In the FET group, either D3 blastomere or D5/6 blastocyst embryo was thawed and transferred with programmed estrogen/progesterone uterine replacement 2 months after oocyte retrieval. In both groups, the luteal phase was supported by transvaginal micronized P, 200 mg three times daily (Uterogestan; Besins Iscovesco, Paris, France) or P in oil, 100 mg IM.

### Follow-up

Serum hCG was measured 14 days after embryo transplantation. Transvaginal ultrasound was performed to determine intrauterine pregnancy, gestational sac number, and embryo heart activity at 7 weeks if the serum hCG test is positive. Telephone interviews were performed to collect live birth data.

### Evaluated parameters

For the patients included in the study, the following characteristics were recorded: age, infertility type, duration of infertility, previous pregnancy, previous delivery, previous miscarriage, body mass index (BMI), basal serum FSH, LH, E_2_, T, and AMH levels. Parameters associated with ovulation stimulation and ovarian response were recorded as following: controlled ovarian hyperstimulation protocol, gonadotrophin usage day, total dose of gonadotrophin, number of oocytes retrieved. Evaluated parameters associated with oocyte developmental competence including: metaphase II (MII) oocyte number, oocyte maturation rate, fertilization rate, two pronuclei cleavage number (2pn), 2pn cleavage rate, day 3 embryo number, day 3 good embryo number (day 3 good embryo defined as 6 to 10 cell blastomere on day 3, < 20% fragmentation, no multi-nucleation), blastocyst formation rate, transfer, and freezing embryo number. Parameters associated with embryo transfer included embryo number, clinical pregnancy rate, implantation rate, clinical pregnancy rate, and live birth rate. All the above parameters were evaluated in association with mural granulosa cells, cumulus, and follicular fluid hormonal levels.

### Statistical analyses

All statistical analyses were performed using SPSS 18.0 software (SPSS Inc., USA). Descriptive parameters and patient characteristics were reported as mean ± standard deviation (SD) or median (range) values depending on variable distribution. Comparisons were made by using the independent sample *t* test for normal distribution variables and were compared using nonparametric Mann-Whitney *U* test for non-normal distribution variables. Chi square test and correlation analysis were used. Correlation coefficients (*r*) and the exact *p* values were calculated. A difference with *P* < 0.05 (two-sided) was considered statistically significant.

## Results

### Information of patients

Characteristics of patients from normal ovarian reserve and diminished ovarian reserve group are shown in Table 1. There were significant differences in age, previous history of pregnancies, deliveries, and miscarriage between two groups. BMI, basal serum FSH, LH, T, AMH, and antral follicle count. FF AMH, E_2_, and P levels also show differences between two groups, with poorer outcomes in the diminished ovarian reserve group.

**Table 1:** Comparison of variable parameters between NOR and DOR group

Parameters	NOR (*n* = 118)	DOR (*n* = 46)	*P* value
Age (year)	29 (27–32)	40 (32.75–43)	< 0.0001
Duration of infertility (year)	3 (2–5)	2.5 (1–5.25)	*ns*
Number of pregnancies(*n*)	0 (0–1)	1.5 (0.75–2)	< 0.0001
Number of deliveries (*n*)	0 (0–0)	0 (0–1)	< 0.0001
Previous miscarriage (*n*)	0 (0–1)	1 (0–1.25)	< 0.0001
BMI (kg/m^2^)	21.00 (19.57–23.16)	23.04 (20.38–24.96)	< 0.005
Basal serum FSH (mIU/mL)	6.39 (5.69–7.34)	8.61 (6.93–10.75)	< 0.0001
Basal serum E2 (pmol/L)	38.10 (28.70–47.10)	37.1 (23.4–53.1)	*ns*
Basal serum LH (mIU/mL)	5.56 (4.09–7.76)	4.47 (3.09–6.33)	< 0.05
Basal serum T (nmol/L)	0.28 (0.20–0.36)	0.22 (0.11–0.29)	< 0.05
Basal serum AMH (ng/mL)	4.94 (3.20–7.59)	0.73 (0.34–1.09)	< 0.0001
AFC	14 (9–20)	4 (2–5)	< 0.0001

All parameters were shown as median (interquartile range (IQR)). *P* < 0.05 (two-sided) was considered statistically significant, Mann-Whitney *U* test was used

*BMI* body mass index, *AFC* antral follicle count

### Apoptosis of mural granulosa cells, cumulus cells, and follicular fluid hormones

The percentages of necrosis, early apoptosis, late apoptosis, and total apoptosis rate of mural granulosa cells and cumulus cells in relation to the normal ovarian reserve or diminished ovarian reserve group are shown in Table 2. The median of early, late, and total apoptosis rate in normal ovarian reserve group were 1.20% (0.42–3.72%), 1.71% (0.37–5.09%), and 4.68% (1.62–10.30%), respectively. The median of early, late, and total apoptosis rate in diminished ovarian reserve group were 2.16% (1.25–8.12%), 5.24% (1.54–9.79%), and 9.67% (4.85–16.89%), respectively. Apoptosis of mural granulosa cells including early, late, and total apoptosis was all increased in diminished ovarian reserve group when compared with normal ovarian reserve group (*P* < 0.01 (two-sided), Mann-Whitney *U* test). While there were no differences in total cumulus cell apoptosis between normal ovarian reserve and diminished ovarian reserve, follicular fluid AMH in normal ovarian reserve group and diminished ovarian reserve were 3.48 ng/mL (2.06–5.84) and 1.42 ng/mL (0.98–2.43). Follicular fluid P in normal ovarian reserve group and diminished ovarian reserve were 18,900 ng/mL (12945–45,380) and 13,800 ng/mL (10350–16,950). Follicular fluid E2 in normal ovarian reserve group and diminished ovarian reserve were 1,628,100 ng/mL (478,044–872,500) and 893,500 ng/mL (614,310–1,085,000), respectively. Follicular fluid AMH (*P* < 0.0001) and P (*P* < 0.05) were significantly decreased while follicular fluid E_2_ (*P* < 0.05) were increased in diminished ovarian reserve group.

**Table 2:** Comparison of apoptosis of MGCs, CCs and FF hormones between NOR and DOR group

Parameters	NOR (*n* = 118)	DOR (*n* = 46)	*P* value
MGCs necrosis rate (%)	3.66 (0.70, 9.94)	5.15 (1.19, 13.52)	ns
MGCs early apoptosis rate (%)	0.49 (0.22–2.32)	1.26 (0.48–1.80)	< 0.0001
MGCs late apoptosis rate (%)	1.96 (0.28–5.59)	4.88 (2.02–9.98)	< 0.001
MGCs total apoptosis rate (%)	2.67 (0.87–6.63)	5.55 (3.80–11.39)	< 0.0001
CCs necrosis rate (%)	1.14 (0.084–1.86)	1.07 (0.44–2.10)	*ns*
CCs early apoptosis rate (%)	0.23 (0.045–0.72)	0.29 (0.20–0.60)	*ns*
CCs late apoptosis rate (%)	4.27 (0.72–13.40)	6.63 (2.69–11.80)	*ns*
CCs total apoptosis rate (%)	5.60 (1.44–14.42)	6.98 (3.12–11.99)	*ns*
FF AMH (ng/mL)	3.48 (2.06–5.84)	1.42 (0.98–2.43)	< 0.0001
FF E2 (pmol/L)	628,100 (478,044–872,500)	893,500 (614,310–1,085,000)	< 0.05
FF P (ng/mL)	18,900 (12,945–45,380)	13,800 (10,350–16,950)	< 0.05
FF T (nmol/L)	5.30 (4.00–8.40)	5.99 (4.26–8.34)	*ns*

All parameters were shown as median (interquartile range (IQR)). *P* < 0.05 (two-sided) was considered statistically significant, Mann-Whitney *U* test was used

### Ovarian response-related results, laboratory results, and clinical outcomes

Parameters associated with ovulation stimulation, ovarian response, laboratory results, and clinical outcomes are shown in Table 3. Parameters associated with ovulation stimulation and ovarian response such as gonadotrophin usage day (7 vs 11; diminished ovarian reserve vs normal ovarian reserve, *P* < 0.0001), total dose of gonadotrophin (1200 vs 1650; diminished ovarian reserve vs normal ovarian reserve, *P* < 0.05), and number of oocytes retrieved (3 vs 14; diminished ovarian reserve vs normal ovarian reserve, *P* < 0.001) were significantly decreased in diminished ovarian reserve group. Laboratory results including 2pn number (1 vs 8; diminished ovarian reserve vs normal ovarian reserve, *P* < 0.001), 2pn cleavage number (1 vs 8; diminished ovarian reserve vs normal ovarian reserve, *P* < 0.001), D3 embryo number (1 vs 6; diminished ovarian reserve vs normal ovarian reserve, *P* < 0.001), D3 good embryo number (1 vs 5; diminished ovarian reserve vs normal ovarian reserve, *P* < 0.001), D3 good embryo rate (67.54% vs 33.33%; diminished ovarian reserve vs normal ovarian reserve, *P* < 0.001), and freezing embryo number (0 vs 3; diminished ovarian reserve vs normal ovarian reserve, *P* < 0.0001) were lower in diminished ovarian reserve . However, there were no differences of fertilization rate, 2pn rate, and blastocyst formation rate between two groups. There were significant differences of clinical pregnancy rate (52.83% vs 27.27%, *P* < 0.0001) and live birth rate (42.45% vs 27.27%, *P* < 0.0001) between normal ovarian reserve and diminished ovarian reserve (patients with normal ovarian reserve vs patients with diminished ovarian reserve), but there was no difference in implantation rate between these two groups.

**Table 3:** Comparison ovarian response-related parameters, laboratory results, and clinical outcomes between NOR and DOR group

Parameters	NOR (*n* = 118)	DOR (*n* = 46)	*P* value
GN day (day)	11 (9–12)	7 (5–10)	< 0.0001
Total GN dosage (IU)	1650 (1293.75–2200)	1200 (750–2569)	< 0.05
OPU egg number	14 (9–19)	3 (1–4)	< 0.001
2pn number	8 (5–12.25)	1 (0.25–1.00)	< 0.001
Fertilization rate	68.42 (57.14–82.79)	75.00 (25.00–100.00)	ns
2pn cleavage number	8 (5–12)	1 (1–3)	< 0.001
2pn cleavage rate (%)	90.00 (75.74–100.00)	79.17 (18.75–100.00)	ns
D3 embryo number	6 (3–10)	1 (0–2)	< 0.001
D3 good embryo number	5 (2.75–8)	1 (0–1.25)	< 0.001
D3 good embryo rate (%)	67.54 (50.00–83.99)	33.33 (0.00–100.00)	< 0.001
Culture D3 embryo number	6 (4–9)	2 (1–3)	< 0.05
Blastocyst number	3 (2–6)	1 (0–2)	ns
Good blastocyst number	2 (1–6)	0 (0–1.75)	ns
Blastocyst formation rate (%)	55.56 (33.33–75.00)	41.67 (0–65.91)	ns
Frozen embryo number	3 (1.75–7)	0 (0–1)	< 0.0001
Embryo transfer *n* (%)	106 (89.83)	22 (48.89)	< 0.0001
Embryo implantation *n* (%)	102 (51.51)	13 (30.23)	ns
Clinical pregnancy *n* (%)	56 (52.83)	6 (27.27)	< 0.0001
Live birth *n* (%)	45 (42.45)	6 (27.27)	< 0.0001

All parameters were shown as median (interquartile range (IQR) and were compared using Mann-Whitney *U* test. The numerical data were expressed as *n* (%) and were compared using Chi-square test or Fisher’s exact test. A difference with *P* < 0.05 (two-sided) was considered statistically significant

*GN day* gonadotrophin usage day, *total GN dosage* total dose of gonadotrophin, *OPU egg number* number of oocytes retrieved

### Correlation analysis of apoptosis of mural granulosa cells and cumulus cells

Total apoptosis of mural granulosa cells were negatively related with hCG day E2 (*r* = − 0.405, *P* < 0.001), gonadotrophin usage day (*r* = − 0.205, *P* < 0.01), antral follicle count (*r* = − 0.240, *P* < 0.01), number of oocytes retrieved (*r* = − 0.361, *P* < 0.0001), number of MII oocytes (*r* = − 0.427, *P* < 0.001), 0pn number (*r* = − 0.281, *P* < 0.0001), 2pn number (*r* = − 0.281, *P* < 0.0001), 2pn cleavage number (*r* = − 0.284, *P* < 0.0001), D3 embryo number (*r* = − 0.277, *P* < 0.0001), D3 good embryo number(*r* = − 0.263, *P* < 0.0001), and freezing embryo number (*r* = − 0.333, *P* < 0.0001). The lower the apoptosis rate of the mural granulosa cells, the greater was the ovarian reserve and clinical outcome. Total apoptosis of mural granulosa cells were positively related to age (*r* = 0.251, *P* < 0.001) and day 3 FSH (*r* = 0.222, *P* < 0.01).

### Apoptosis of mural granulosa cells, cumulus cells, and follicular fluid hormones in different age groups

Since we found a positive correlation between mural granulosa cell apoptosis rate and age, we further analyzed the apoptosis and follicular fluid hormone results in different age groups (Table 4). The percentages of early apoptosis, late apoptosis, and total apoptosis rate of mural granulosa cells were significantly increased in women age ≥ 37 years old. Follicular fluid AMH was significantly decreased in women age ≥ 37 years old.

**Table 4:** Comparison of apoptosis of MGCs, CCs, and FF hormones in different age groups

Parameters	< 30 (*n* = 64)	30–37 (*n* = 70)	≥ 37 (*n* = 30)	*P* value
MGCs necrosis rate (%)	5.33(1.00–15.40))	3.51 (0.55–10.29)	3.61 (0.75–8.40)	*ns*
MGCs early apoptosis rate (%)	0.45 (0.20–1.00)	0.62 (0.25–1.46)	1.30 (0.535–2.40)	< 0.0001
MGCs late apoptosis rate (%)	2.08 (0.29–5.67)	2.26 (0.42–4.81)	6.40 (2.33–15.70)	< 0.05
MGCs total apoptosis rate (%)	2.85 (0.83–6.42)	2.77 (1.00–6.69)	6.91 (3.92–17.05)	< 0.0005
CCs necrosis rate (%)	1.24 (0.20–2.01)	0.49 (0.06–1.70)	1.07 (0.44–2.17)	*ns*
CCs early apoptosis rate (%)	0.21 (0.04–0.66)	0.31 (0.10–1.06)	0.30 (0.20–1.03)	*ns*
CCs late apoptosis rate (%)	4.46 (0.78–13.75)	5.83 (0.72–11.8)	5.76 (2.65–10.52)	*ns*
CCs total apoptosis rate (%)	5.60 (1.77–15.00)	6.79 (1.44–12.18)	6.80 (3.07–10.90)	*ns*
FF AMH (ng/mL)	3.66 (2.33–6.22)	2.57 (1.44–4.30)	1.77 (0.80–2.66)	< 0.05
FF E2 (pmol/L)	692,885 (491,558–988,625)	618,862 (470,400–893,500)	816,500 (615,000–1,157,250)	*ns*
FF P_4_ (ng/mL)	21,010 (14,100–46,000)	15,000 (12,000–29,990)	14,400 (9750–17,100)	*ns*
FF T (nmol/L)	5.80 (4.27–8.66)	5.18 (3.99–11.85)	5.99 (3.63–8.34)	*ns*

All parameters were shown as median (interquartile range (IQR)). *P* < 0.05 (two-sided) was considered statistically significant, Kruskal-Wallis *H* test was used

### Comparison of parameters between clinical pregnant and non-pregnant group

The cumulus cells early apoptosis rate in the clinical pregnant group and the non-pregnant group were 0.13% (0.032–0.86%) and 0.37% (0.19–0.69%), respectively (Table 5). Follicular fluid E_2_ in the pregnant group and non-pregnant group were 602,497 pmol/L (468,066–835,750) and 716,750 pmol/L (0.19–0.69%), respectively. The cumulus cells early apoptosis rate and follicular fluid E_2_ were significantly higher in non-pregnant group compared with the clinical pregnant group. Good blastocyst number and blastocyst formation rate were significantly higher in the clinical pregnant group.

**Table 5:** Comparison of parameters between clinical pregnant and non-pregnant group

Parameters	Clinical pregnant (*n* = 62)	Non-pregnant (*n* = 66)	*P* value
Age (year)	29 (27.75–32)	30 (27–33)	*ns*
Duration of Infertility (year)	3 (2–4)	3 (2–5)	*ns*
Number of pregnancies(*n*)	1 (0–1)	1 (0–1)	*ns*
Number of deliveries (*n*)	0 (0–0)	0 (0–0)	*ns*
Previous miscarriage (*n*)	0 (0–1)	1 (0–1)	*ns*
BMI (kg/m^2^)	21.37 (19.99–23.77)	20.84 (19.08–23.01)	*ns*
Basal serum FSH (mIU/mL)	6.57 (5.36–7.35)	6.62 (5.73–7.55)	*ns*
Basal serum E2 (pmol/L)	37.3 (28.55–47.7)	37.1 (26.3–44.3)	*ns*
Basal serum LH (mIU/mL)	5.52 (4.03–7.54)	5.26 (3.96–8.39)	*ns*
Basal serum T (nmol/L)	0.28 (0.20–0.36)	0.27 (0.19–0.35)	*ns*
Basal serum AMH (ng/mL)	4.34 (2.14–7.46)	3.76 (1.62–6.03)	*ns*
AFC	13.5 (8–20)	10.5 (6–16)	0.062
MGCs necrosis rate (%)	7.29 (0.65–11)	4.06 (1.00–14.8)	*ns*
MGCs early apoptosis rate (%)	0.58 (0.29–1.08)	0.63 (0.22–1.45)	*ns*
MGCs late apoptosis rate (%)	2.75 (0.67–6.31)	2.36 (0.06–5.68)	*ns*
MGCs total apoptosis rate (%)	3.33 (1.04–7.69)	3.28 (0.74–6.69)	*ns*
CCs necrosis rate (%)	0.87 (0.084–1.73)	1.28 (0.23–2.36)	*ns*
CCs early apoptosis rate (%)	0.13 (0.032–0.86)	0.37 (0.19–0.69)	< 0.05
CCs late apoptosis rate (%)	2.46 (0.47–13.4)	6.63 (1.36–14.0)	*ns*
CCs total apoptosis rate (%)	2.63 (0.56–14.42)	8.71 (3.81–14.61)	0.052
FF AMH (ng/mL)	3.33 (1.83–4.98)	2.91 (1.14–5.28)	*ns*
FF E2 (pmol/L)	602,497 (468,066–835,750)	716,750 (612,033–942,250)	< 0.05
FF P (ng/mL)	17,700 (12,300–45,935)	17,700 (12,697.5–29,052.5)	*ns*
FF T (nmol/L)	5.26 (3.5–8)	5.29 (4.38–8.48)	*ns*
GN day (day)	11 (9–12)	10 (9–12)	*ns*
Total GN Dosage (IU)	1650 (1275–2200)	1650 (1200–2287.5)	*ns*
hCG day E_2_(pmol/L)	4759 (2943–7258.5)	2691 (1267–4812)	0.069
OPU egg number	13 (8–17.25)	10.5 (6–18)	*ns*
MII egg number	9 (5.5–13.5)	6 (3–13)	*ns*
2pn number	8 (5–11.25)	6 (3.75–11)	*ns*
Fertilization rate	75.00 (60.00–83.93))	67.26 (57.14–83.33)	*ns*
2pn cleavage number	8 (5–11)	6 (3.75–11)	*ns*
2pn cleavage rate (%)	90.69 (78.37–100)	93.30 (75–100)	*ns*
D3 embryo number	6 (4–9)	5 (2–9)	*ns*
D3 good embryo number	5.5 (23–8)	4 (2–6.25)	0.050
D3 good embryo rate (%)	70.71 (59.29–94.23)	70.98 (41.25–83.55)	*ns*
Culture D3 embryo number	6 (4–9)	4 (2–9)	*ns*
Blastocyst number	3 (2–6)	3 (1–5)	*ns*
Good blastocyst number	3 (1–5)	2 (0–3.25)	< 0.05
Blastocyst formation rate (%)	55.56 (50.00–83.33)	41.67 (17.21–71.63)	< 0.05
Freezing embryo number	4 (2–6.25)	2 (0.5–5.5)	0.08

All parameters were shown as median (p25, p75). *P* < 0.05 (two-sided) was considered statistically significant, Mann-Whitney *U* test was used

*GN day* gonadotrophin usage day, *total GN dosage* total dose of gonadotrophin, *OPU egg number* number of oocytes retrieved

## Discussion

Apoptosis of mural granulosa cells and cumulus cells are different in patients with diminished vs normal ovarian reserve. There is significantly less mural granulosa cell apoptosis with normal ovarian reserve. Likewise with diminished ovarian reserve, there is much greater mural granulosa cell apoptosis. Interestingly, there is no such difference in total cumulus cell apoptosis, but the early apoptosis rate is also correlated with poorer outcomes when comparing the clinical pregnant group to non-pregnant group. This may be the first paper to compare mural granulosa cells to cumulus cells in patients with diminished or normal ovarian reserve. Clearly, as the ovary ages, the poorer results with IVF/ICSI can be correlated with increased apoptosis of the mural granulosa cells. One could speculate that the aging of the oocyte and the aging of the somatic supporting cells are related.

Apoptosis, or programmed cell death, is a normal physiologic process for removal of unwanted cells [17]. One of the earlier events of apoptosis includes translocation of membrane phosphatidylserine (PS) from the inner side of the plasma membrane to the surface. Annexin V, a calcium-dependent phospholipid-binding protein, has high affinity for PS; therefore, fluorescently labeled annexin V can be used for the detection of exposed PS using flow cytometry [18]. By co-staining with propidium iodide (PI) and analyzing with flow cytometry, cell population can be further classified as necrotic cells (annexin V-PI+), early apoptotic cells (annexin V + PI−), late apoptotic cells (annexin V + PI+), and viable cells (annexin V − PI−). Annexin V/PI staining is one of the most commonly used assays to measure apoptosis and necrosis [17, 18]. Other methods for measuring programmed cell death include array transcriptome analysis, and autophagy, and mitophagy, but are not included in this paper.

Diminished ovarian reserve is a term related to both oocyte quantity and quality [19, 20]. However, it is much easier and direct to measure oocyte yield than oocyte quality in diminished ovarian reserve patients in clinical practice. Our study shows higher mural granulosa cell apoptosis including early, late, and total apoptosis, associated with diminished ovarian reserve. Also, mural granulosa cell apoptosis was negatively correlated with parameters associated with ovarian stimulation and ovarian response, including hCG day E2, gonadotrophin usage day, number of oocytes retrieved.

Mural granulosa cell apoptosis when combined with age and basal serum AMH can be used as a marker to predict number of oocytes retrieved in a linear regression model (*R* = 0.702, *P* < 0.005) (Supplemental Table 1). Less mural granulosa cell apoptosis is associated with better ovarian response and higher oocyte yield in IVF/ICSI.

The question of whether mural granulosa cell apoptosis has direct relationship with oocyte quality is much more difficult to answer in the studies thus far reported. Sadraie et al. [21] found a significantly higher incidence of apoptotic cells in mural granulosa cells in women ˃ 40 years old with less oocytes retrieved and less mature oocytes. Corn et al. [38] reported that apoptotic processes seem to impair oocyte maturation and lower the chance of blastocyst formation. However, Jancar et al. [23] reported that apoptosis and reactive oxygen species production in granulosa cells have no significant impact on fertilization and do not correlate with the development of blastocysts. In our study, we found that there is a higher percentage of total apoptotic cells in mural granulosa cells in women ≥ 37 years old, which is consistent with Sadraie and Corn’s results. Mural granulosa cell apoptosis is negatively correlated to the number of MII oocytes, number of 2pn cleavage, number of D3 embryos, number of D3 good embryos, and number of frozen embryos. The lower the apoptosis rate of the mural granulosa cells, the greater is the mature oocyte and embryo yield. Mural granulosa cell apoptosis might possibly be not only correlated with ovarian response, but also might correlate with oocyte quality and subsequent embryo development.

There is an elaborate and complex junctional communication system which mediates the information flow between cumulus granulosa cells and oocyte within the ovarian follicle during oocyte maturation [24]. Studies have shown that mitochondrial characteristics of cumulus cells may serve as indicators of oocyte competence [25] and embryo quality [25, 26]. Transcripts and microarray experiments of cumulus cell can be used as markers of oocyte and embryo quality [27] and used in single embryo selection to increase live birth rate [28]. We found a significant increase of total mural granulosa cell apoptosis in women with diminished ovarian reserve and a negative correlation with oocyte yield, embryo development, and clinical outcome. We did not observe a significant difference of total cumulus cell apoptosis in diminished ovarian reserve when compared with normal ovarian reserve. One explanation may be that the granulosa cells are more susceptible to apoptosis in the follicle than the theca or cumulus cells [37]. In a recent paper about microRNA in young women with diminished ovarian reserve, only nine miRNAs were found to be differentially expressed in cumulus cells in young women with diminished ovarian reserve, while there were 105 miRNA differentially expressed in granulosa cells [16]. However, when we compared parameters between the clinical pregnant group and the non-pregnant group, cumulus cell early apoptosis rate was significantly lower in the pregnant group.

There were similar baseline factors including age, basal serum AMH, gonadotropin dosage, stimulation days, and retrieval oocyte number between pregnant and non-pregnant group. Higher number of D3 good embryo, good blastocyst, and blastocyst formation rate were observed in pregnant group. Even though the percentage of early apoptosis rate of cumulus cells was relatively low in both groups, it may be a sensitive marker in cumulus cell quality. When adjusting the influence of ovarian response and ovarian stimulation, early cumulus cell apoptosis might have an influence on embryo development and pregnancy.

We found that follicular fluid E_2_ level was significantly higher while follicular fluid P4 and AMH level was lower in diminished ovarian reserve group. Estrogens and progesterone represent the key ovarian hormones produced by the developing ovulatory follicle [29]. In our study, we found that follicular fluid contained much higher levels of E_2_ and P_4_ 34–36 h after hCG trigger, consistent with other studies [30]. In follicular fluid, E_2_ was a negative predictor of the number of oocytes retrieved and the number of day 5 blastocysts [30]. Intrafollicular AMH levels were positively related to P4 [31]. After hCG trigger, there is an upregulation of P_4_ and downregulation of estradiol level in serum. Higher levels of estradiol and lower levels of progesterone in follicular fluid in women with diminished ovarian reserve may indicate a dysfunction and poor response to hCG trigger, resulting in a failure of transition between E_2_ and P4.

One of the deficiencies in our study is that patients were assigned to different stimulation protocols for controlled ovarian hyperstimulation according to physician’s preference. Previous studies have shown that controlled ovarian hyperstimulation protocols may affect apoptosis in cumulus cells [22, 32–35]. This may influence the apoptosis and clinical outcomes between diminished ovarian reserve and normal ovarian reserve. However, we did not observe a significant difference in granulosa cell apoptosis and follicular fluid hormones among different controlled ovarian hyperstimulation protocols in this prospective cohort (Supplemental Table 2). Thus, we combined data from different controlled ovarian hyperstimulation protocols in our analysis.

Another deficiency is that we needed to pool together the follicle fluid to isolate mural granulosa cells. It was not available to us to correlate each follicle with oocyte and embryo development [36]. But we still were able to analyze the overall mural granulosa cell apoptosis rate in women with diminished ovarian reserve. Thus, we were able to compare both mural granulosa cells and cumulus cells, with follicular fluid hormones in each patient.

Perhaps the most important deficiency in this report is that we studied only one of the many pathways to programmed cell death, i.e., annexin V-FITC/PI apoptosis staining and flow cytometry. In order to determine more clearly the relationship of apoptosis of mural and cumulous cells to ovarian reserve and egg quality, these pathways will also need to be studied.

In conclusion, according to our data, total mural granulosa cell apoptosis (but not total cumulus cell apoptosis) is increased in women with diminished ovarian reserve, correlated with worse ovarian response, and with fewer egg and embryo numbers. Mural granulosa cell apoptosis is increased in women ≥ 37 years old. Mural granulosa cell apoptosis may be used as a marker to indicate oocyte yield and ovarian response in women undergoing IVF/ICSI. Direct relationship between granulosa cell/cumulus cell apoptosis and oocyte quality will need to be investigated in future studies. Furthermore, other pathways and methodologies of cell death in mural and granulosa cells will need to be studied.

## Electronic supplementary material

ESM 1 (DOCX 15 kb)

ESM 2 (DOCX 16 kb)
