# Supplementary material for: Apoptosis of mural granulosa cells is increased in women with diminished ovarian reserve
Source: J Assist Reprod Genet. 2019 Apr 13;36(6):1225–35. doi: 10.1007/s10815-019-01446-5 (PMC6602993; doi:10.1007/s10815-019-01446-5)
Supplement: Supplementary file 1 — (DOCX 15 kb) [file 10815_2019_1446_MOESM1_ESM.docx]

Supplemental Table 1 linear regression of number of oocyte retrieval

| **Model Summary** | | | | |
| --- | --- | --- | --- | --- |
| Model | R | R Square | Adjusted R Square | Std. Error of the Estimate |
| 1 | .702^a^ | .492 | .483 | 5.490 |
| a. Predictors: (Constant), basic_AMH, GCQ2+3, age | | | | |

| **ANOVA^a^** | | | | | | |
| --- | --- | --- | --- | --- | --- | --- |
| Model | | Sum of Squares | df | Mean Square | F | Sig. |
| 1 | Regression | 4559.761 | 3 | 1519.920 | 50.433 | .000^b^ |
|  | Residual | 4701.433 | 156 | 30.137 |  |  |
|  | Total | 9261.194 | 159 |  |  |  |
| a. Dependent Variable: OPU_egg | | | | | | |
| b. Predictors: (Constant), basic_AMH, GCQ2+3, age | | | | | | |

| **Coefficients^a^** | | | | | | | | | | |
| --- | --- | --- | --- | --- | --- | --- | --- | --- | --- | --- |
| Model | | Unstandardized Coefficients | | Standardized Coefficients | t | Sig. | 95.0% Confidence Interval for B | | Collinearity Statistics | |
|  |  | B | Std. Error | Beta |  |  | Lower Bound | Upper Bound | Tolerance | VIF |
| 1 | (Constant) | 24.349 | 3.059 |  | 7.961 | .000 | 18.308 | 30.391 |  |  |
|  | Age (yr) | -.484 | .088 | -.352 | -5.485 | .000 | -.659 | -.310 | .792 | 1.263 |
|  | MGCs total apoptosis (%) | -.124 | .041 | -.178 | -3.015 | .003 | -.206 | -.043 | .937 | 1.067 |
|  | Basal serum AMH (ng/mL) | .842 | .127 | .414 | 6.625 | .000 | .591 | 1.092 | .835 | 1.198 |
| a. Dependent Variable: number of oocyte retrieval | | | | | | | | | | |

| **Collinearity Diagnostics^a^** | | | | | | | |
| --- | --- | --- | --- | --- | --- | --- | --- |
| Model | Dimension | Eigenvalue | Condition Index | Variance Proportions | | | |
|  |  |  |  | (Constant) | age | GCQ2+3 | basic_AMH |
| 1 | 1 | 3.145 | 1.000 | .00 | .00 | .03 | .02 |
|  | 2 | .559 | 2.372 | .00 | .00 | .56 | .25 |
|  | 3 | .285 | 3.324 | .01 | .02 | .38 | .49 |
|  | 4 | .011 | 17.049 | .99 | .98 | .02 | .24 |
| a. Dependent Variable: OPU_egg | | | | | | | |
