# Supplementary material for: Apoptosis of mural granulosa cells is increased in women with diminished ovarian reserve
Source: J Assist Reprod Genet. 2019 Apr 13;36(6):1225–35. doi: 10.1007/s10815-019-01446-5 (PMC6602993; doi:10.1007/s10815-019-01446-5)
Supplement: Supplementary file 2 — (DOCX 16 kb) [file 10815_2019_1446_MOESM2_ESM.docx]

**Supplemental Table 2**: Comparison of apoptosis of MGCs, CCs and FF hormones between different COH protocols

| Parameters | Antagonist protocol  (*n* = 49) | Long protocol  (*n* = 86) | Mini stimulation  (*n* = 29) | *df* | *P* value |
| --- | --- | --- | --- | --- | --- |
| MGCs necrosis rate (%) | 3.16 (0.605-5.61) | 7.00 (1.37-15.35) | 3.40 (0.35-10.60) | *284* | *0.555* |
| MGCs early apoptosis rate (%) | 0.63 (0.23-1.44) | 0.49 (0.23-1.13) | 1.33 (0.48-2.30) | *240* | *0.526* |
| MGCs late apoptosis rate (%) | 3.12 (0.34-6.90) | 2.13 (0.30-5.72) | 3.78(2.00-15.10) | *272* | *0.466* |
| MGCs total apoptosis rate (%) | 4.22 (0.82-7.30) | 2.66 (0.96-6.57) | 5.11 (3.33-16.54) | *288* | *0.362* |
| CCs necrosis rate (%) | 0.91 (0.42-1.70) | 1.28 (0.15-2.25) | 1.04 (0.50-1.80) | *174* | *0.380* |
| CCs early apoptosis rate (%) | 0.19 (0.035-0.38) | 0.38 (0.11-0.83) | 0.30 (0.20-1.05) | *160* | *0.762* |
| CCs late apoptosis rate (%) | 2.42 (0.20-9.29) | 6.89 (2.04-14.00) | 6.63 (2.78-11.21) | *188* | *0.423* |
| CCs total apoptosis rate (%) | 3.23 (0.50-11.51) | 8.74 (2.63-18.19) | 6.98 (3.02-11.64) | *204* | *0.470* |
| FF AMH (*n*g/mL) | 5.22 (2.92-8.27) | 2.62 (1.47-3.88) | 2.23 (1.44-4.01) | *180* | *0.318* |
| FF E2 (*p*mol/L) | 724500 (458369.0-912733.5) | 643750 (510560.5-910625.0) | 763000 (474326.5-1027224.0) | *200* | *0.408* |
| FF P (*n*g/mL) | 14000 (10200-31740) | 18300 (13950-45925) | 13200 (10800-15600) | *158* | *0.492* |
| FF T (*n*mol/L) | 7.02 (4.23-8.53) | 5.17 (4.00-7.94) | 8.44 (4.90-18.02) | *156* | *0.740* |

All Parameters were shown as median (interquartile range (IQR)). *P* <0.05 (two-sided) was considered statistically significant, Chi square test was used.
